# FuSeBMC: A White-Box Fuzzer for Finding Security Vulnerabilities in C Programs (Competition Contribution)

**DOI:** 10.1007/978-3-030-71500-7_19

**Published:** 2021-02-24

**Authors:** Kaled M. Alshmrany, Rafael S. Menezes, Mikhail R. Gadelha, Lucas C. Cordeiro

**Affiliations:** 8 Universidad Autónoma de Madrid, Madrid, Spain; 9 University of Twente, Enschede, The Netherlands; 10 University of Manchester, Manchester, UK Institute of Public Administration, Jeddah, Saudi Arabia; 11 Federal University of Amazonas, Manaus, Brazil; 12 SIDIA Instituto de Ciência e Tecnologia, Manaus, Brazil; 13 University of Manchester, Manchester, UK

**Keywords:** Automated Test-Case Generation, Symbolic Execution, Bounded Model Checking, Fuzzing, Security.

## Abstract

We describe and evaluate a novel white-box fuzzer for C programs named FuSeBMC, which combines fuzzing and symbolic execution, and applies Bounded Model Checking (BMC) to find security vulnerabilities in C programs. FuSeBMC explores and analyzes C programs (1) to find execution paths that lead to property violations and (2) to incrementally inject labels to guide the fuzzer and the BMC engine to produce test-cases for code coverage. FuSeBMC successfully participates in Test-Comp’21 and achieves first place in the Cover-Error category and second place in the Overall category.

## 1 Test Generation Approach

Automated test-case generation is a method to check whether the software matches expected requirements [2]. It involves the automated execution of software components to evaluate intricate properties and achieve code coverage metrics (e.g., decision, branch, instruction). Here, we describe and evaluate a novel white-box fuzzer, FuSeBMC, capable of automatically producing test-cases for C programs. FuSeBMC provides an innovative software testing framework that detects security vulnerabilities in C programs by using fuzzing and symbolic execution in combination with Bounded Model Checking (BMC) (cf. Fig. 1). FuSeBMC builds on top of clang [1] to instrument the C program, uses Map2check [8] as a fuzzing engine, and ESBMC (Efficient SMT-based Bounded Model Checker) [4,5] as BMC and symbolic execution engines, thus combining dynamic and static verification techniques.

⋆ Jury Member

E. Guerra and M. Stoelinga (Eds.): FASE 2021, LNCS 12649, pp. 363–367, 2021.
https://doi.org/10.1007/978-3-030-71500-7_19

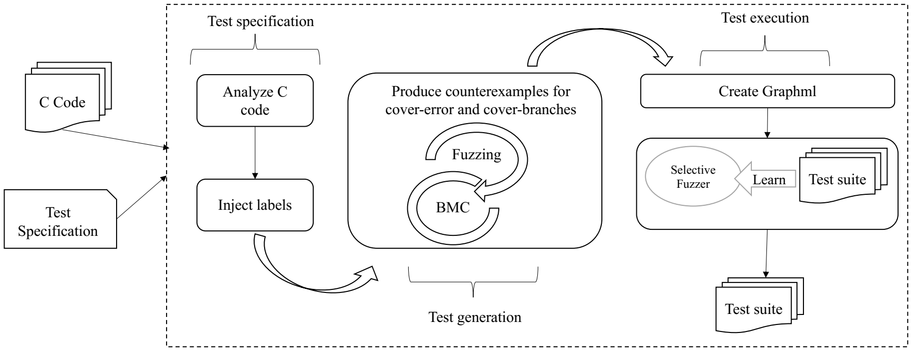

Fig. 1: FuSeBMC: a white-box fuzzer framework for C Programs.

FuSeBMC takes a C program and a test specification [3] as input. In the `Cover-Error` category, FuSeBMC invokes the fuzzing and BMC engines sequentially to find a path that violates a given property. It uses an iterative BMC approach that incrementally unwinds the program until it finds a property violation or exhausts time or memory limits. FuSeBMC uses incremental BMC to explore the program state space searching for a property violation since all programs in Test-Comp'21 are known to have issues. In the `Cover-Branches` category, FuSeBMC explores and analyzes the target C program using the clang compiler to inject labels incrementally. FuSeBMC will compute all branches of the C code and inject the labels for each branch by adding the label `GOAL-`$N$, where $N$ is the goal number. Both engines will check whether these injected labels are reachable to produce test-cases for branch coverage.

FuSeBMC analyzes the counterexamples and saves them as a *graphml* file. It checks whether the fuzzing and BMC engines could produce counterexamples for both categories `Cover-Error` and `Cover-Branches`. If that is not the case, FuSeBMC employs a second fuzzing engine named selective fuzzer which produces test-cases for the rest of the labels. The selective fuzzer produces test-cases by learning from the two engines' output: it analyzes the range of the inputs that should be passed to examine the target C program and then produces different test-cases. Lastly, FuSeBMC prepares valid test-cases with metadata to test a target C program using TestCov [3] as a test validator.

FuSeBMC sets a 150 seconds limit for the fuzzing engine and a 700 seconds limit for the BMC engine and sets a 50 seconds limit for the selective fuzzer. These numbers were obtained empirically by analyzing the Test-Comp'21 results.

## 2 Strengths and Weaknesses

Incremental BMC allows FuSeBMC to keep unwinding the program until a property violation is found or time or memory limits are exhausted. This approach is advantageous in the `Cover-Error` category as finding one error is the primary goal. Another strength of FuSeBMC is that it can accurately model C programs that use the IEEE floating-point arithmetic [6,7]. The floating-point encoding layer in our BMC engine extends the support for the SMT FP theory to solvers that do not support it natively. FuSeBMC can test programs with floating-point arithmetic using all currently supported solvers in BMC engine (ESBMC), including Boolector [9], which does not support the SMT FP theory natively.

In both Cover-Error and Cover-Branches categories, various test-cases produced by FuSeBMC are validated successfully. The majority of our test-cases were produced by the BMC engine and the selective fuzzer; our fuzzing engine did not produce many test-cases because it does not model the C library, so it mostly guesses the inputs. For example, in the Cover-Error category, TestCov confirms 500 test-cases produced by FuSeBMC, where our fuzzing engine produces 13 (Map2Check), BMC engine produces 393 (ESBMC), while our selective fuzzer produces 94 test-cases (selective).

However, note that our fuzzing engine is not limited to only produce test-cases. It helps our selective fuzzer by providing information about the number of inputs required to trigger a property violation, i.e., the number of assignments required to reach an error. In several cases, the BMC engine can exhaust the time limit before providing such information, e.g., when there are large arrays that need to be initialized at the beginning of the program. For example, consider the following code fragment extracted from the standard_copy1_ground-2.c benchmark, as illustrated in Fig. 2.

```
#define N 100000
  ...
  int a, a1[N], a2[N];
  for (a = 0 ; a < N ; a++) {
      a1[a] = __VERIFIER_nondet_int();
      a2[a] = __VERIFIER_nondet_int();
  }
  ...
  for (int x = 0 ; x < N ; x++)
    __VERIFIER_assert(a1[x] == a2[x]);
```

Fig. 2: Code fragment that contains a large array.

In this particular example, ESBMC exhausts the time limit before checking the assertion $a1[x] == a2[x]$. Apart from that, our employed verification engines also demonstrate a certain level of weakness to produce test-cases due to the many optimizations we perform when converting the program to SMT. In particular, two techniques affected the test-case generation significantly: *constant folding* and *slicing*. *Constant folding* evaluates constants (which includes nondeterministic symbols) and propagates them throughout the formula during encoding, and *slicing* removes expression not in the path to trigger a property violation. These two techniques can significantly reduce SMT solving time. However, they can remove the expressions required to trigger a violation when the program is compiled, i.e., variable initialization might be optimized away, forcing FuSeBMC to generate a test-case with undefined behavior.

Regarding our fuzzing engine, we identified a limitation to handle programs with pointer dereferences. The fuzzing engine keeps track of variables throughout the program but has issues identifying when they go out of scope. When we try to generate a test-case that triggers a pointer dereference, our fuzzing engine provides thrash values, and the selective fuzzer might create test-cases that do not reach the error.

## 3 Tool Setup and Configuration

In order to run our `fusebmc.py` script,[5] one must set the architecture (*i.e.*, 32 or 64-bit), the competition strategy (i.e., $k$-induction, falsification, or incremental BMC), the property file path, and the benchmark path, as:

```
fusebmc.py [-a {32, 64}] [-p PROPERTY_FILE]
                    [-s {kinduction,falsi,incr,fixed}]
                    [BENCHMARK_PATH]
```

where `-a` sets the architecture, `-p` sets the property file path, and `-s` sets the strategy (e.g., `kinduction`, `falsi`, `incr`, or `fixed`). For Test-Comp'21, FuSeBMC uses `incr` for incremental BMC.

When choosing the fuzzing engine, we set the following options when executing Map2Check: timeout of 150 seconds for Map2Check in `Cover-Error`, and a timeout of 70 seconds in `Cover-Branches`; `--fuzzer-mb` 1000 limits memory to 1000 MB; `--target-function-name reach−error` defines the function name to be searched; `--target-function` checks whether the target-function is reachable; `--nondet-generator fuzzer` uses only fuzzing; `--generate-witness` sets the witness output path.

By choosing incremental BMC, the following options are set when executing ESBMC: `--no-div-by-zero-check` disables the division by zero check (required by Test-Comp); `--force-malloc-success` sets that all dynamic allocations succeed (a Test-Comp requirement); `--floatbv` enables floating-point SMT encoding; `--incremental-bmc` enables incremental BMC; `--unlimited-k-steps` removes the upper limit of iteration steps for incremental BMC; `--witness-output` sets the witness output path; `--no-bounds-check` and `--no-pointer-check` disable bounds-check and pointer-safety checks, resp., since we are only interested in finding reachability bugs; `--k-step 5` sets the incremental BMC to 5; `--no-allign-check` disables pointer alignment checks; and `--no-slice` disables slicing of unnecessary instructions.

The Benchexec tool info module is named `fusebmc.py` and the benchmark definition file is `FuSeBMC.xml`.

[5] https://gitlab.com/sosy-lab/test-comp/archives-2021/-/blob/master/2021/FuSeBMC.zip

## 4 Software Project

The FuSeBMC source code is written in C++ and it is available for downloading at GitHub,[6] which includes the latest release of FuSeBMC v3.6.6. FuSeBMC is publicly available under the terms of the MIT License. Instructions for building FuSeBMC from the source code are given in the file `README.md` (including the description of all dependencies).
